# Comparison of cardiac computed tomography and transesophageal echocardiography for left atrial appendage thrombus detection

**DOI:** 10.1186/s12872-026-06012-3

**Published:** 2026-05-29

**Authors:** Ahmet Hakan Ateş, Samuray Zakariyayev, Cem Çöteli, Uğur Nadir Karakulak, Mert Doğan, Halil Baris Basarir, Selin Ardalı, Hikmet Yorgun, Necla Özer, Tuncay Hazırolan, Kudret Aytemir

**Affiliations:** 1 Department of Cardiology, Hacettepe University, Sıhhıye, Ankara, 06230 Turkey; 2 Faculty of Medicine, Department of Radiology, Hacettepe University, Ankara, Turkey; 3 Department of Cardiology, Baku Health Center, Baku, Azerbaijan

**Keywords:** Left atrial appendage thrombus, Cardiac computed tomography, Transesophageal echocardiography, Atrial fibrillation

## Abstract

**Background and objective:**

Pulmonary vein isolation (PVI) is a common intervention for symptomatic atrial fibrillation (AF). Transesophageal echocardiography (TEE) and cardiac computed tomography (CCT) are commonly used pre-PVI to assess left atrial (LA) and left atrial appendage (LAA) thrombus and visualize LA and pulmonary vein anatomy. This retrospective study aims to compare CCT and TEE in detecting LAA thrombus in patients with symptomatic paroxysmal or persistent AF scheduled for PVI.

**Methods:**

Seven hundred five patients underwent routine TEE and CCT within 24 h. Patients with prior LAA thrombus, cardioembolic stroke, or contraindications to TEE were excluded. The presence or absence of LAA thrombus and other potential thrombus-inducing factors were evaluated using TEE and CCT, and the diagnostic performance of the two imaging modalities was compared.

**Results:**

Most patients had paroxysmal AF (54.2%) and a high CHA₂DS₂-VASc score (47.6%). TEE detected LAA thrombus in 1.8%, while 7% had SEC without thrombus. Cardiac CT scans showed LAA filling defects in 17.2%. Using TEE as the reference, CCT demonstrated high sensitivity (92.3%) and negative predictive value (99.8%), but low specificity (84.2%) and positive predictive value (9.9%) for LAA thrombus detection.

**Conclusion:**

The study supports CCT as a reliable imaging method for excluding LAA thrombus in AF patients scheduled for PVI. In cases where CCT shows no thrombus and adequate anticoagulation is ensured, catheter ablation and DCCV procedures can be safely considered.

**Graphical abstract:**

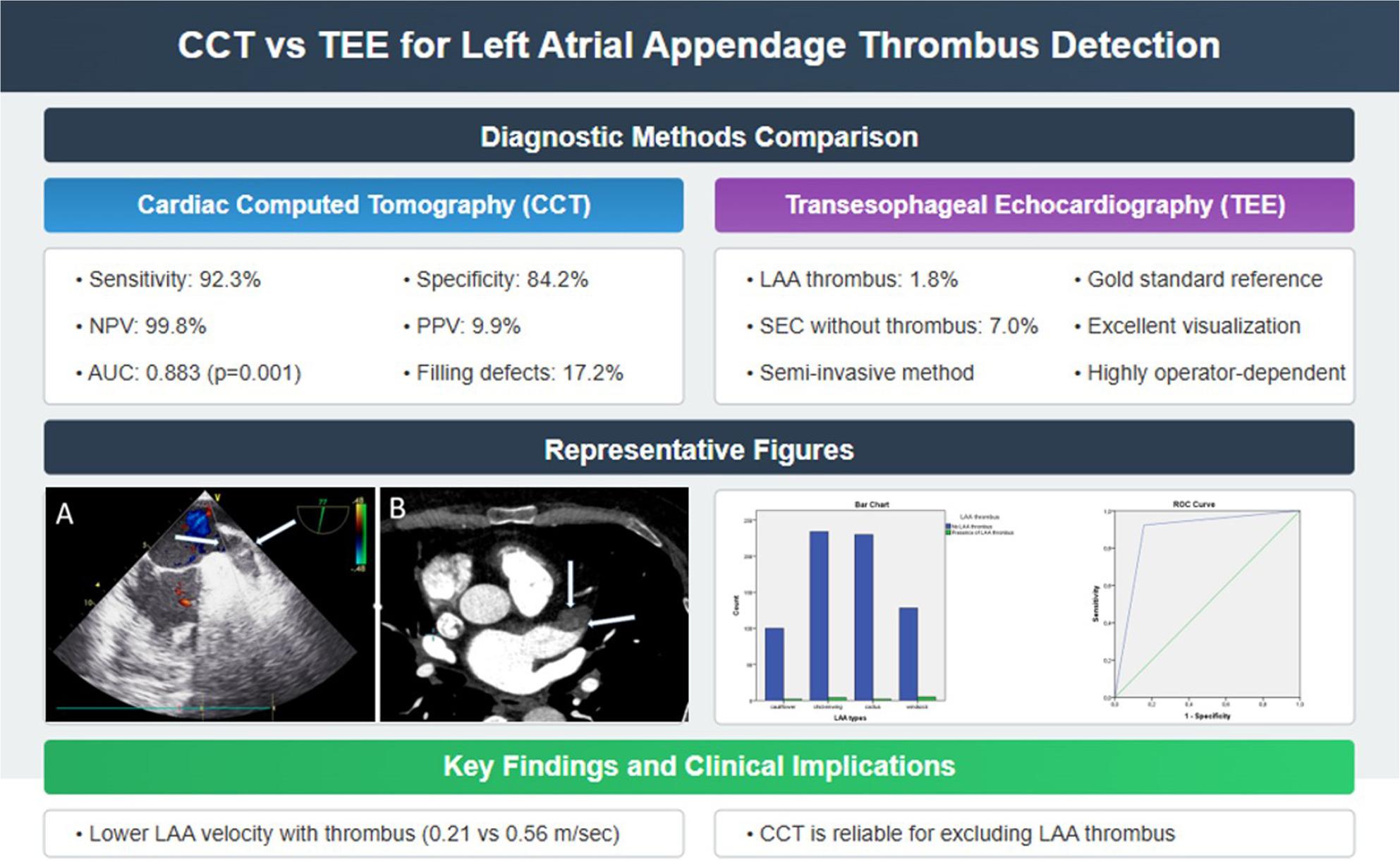

**Supplementary Information:**

The online version contains supplementary material available at 10.1186/s12872-026-06012-3.

## Introduction

Atrial fibrillation (AF) is the most common cardiac arrhythmia in adults worldwide, associated with higher morbidity and mortality rates [1]. Catheter ablation (CA) is an effective treatment for patients with symptomatic AF [2, 3]. However, thromboembolic complications are significant drawbacks of CA. The presence of a thrombus in the left atrium (LA) or left atrial appendage (LAA) increases the risk of thromboembolic complications [4]. Consequently, it is crucial to assess the LA and LAA for thrombus prior to AF ablation. Transesophageal echocardiography (TEE) is the gold standard for LAA thrombus evaluation [5, 6]. Nonetheless, TEE is semi-invasive and time-consuming, with drawbacks like physical discomfort and potentially life-threatening complications [7].

Cardiac Computed Tomography (CCT) is increasingly favored as a primary screening tool because it is non-invasive, thereby maximizing patient comfort, eliminating procedural risks associated with TEE (such as sedation-related complications, esophageal injury, or aspiration), and providing exquisite detailed cardiac anatomy (including 3D pulmonary vein mapping, LAA morphology, and anatomical variants). However, its diagnostic accuracy in comparison to TEE remains debated [8–10].

This study aims to compare CCT and TEE for LAA thrombus evaluation in symptomatic AF patients undergoing PVI.

## Methods

### Study population

We conducted a retrospective analysis of 807 patients with symptomatic paroxysmal or persistent AF who were scheduled for PVI in our hospital between August 2014 and December 2022. All patients underwent standard TEE screening and CCT within a 24-hour timeframe preceding radiofrequency catheter ablation. All patients received effective dosages of anticoagulation therapy in accordance with the recommendations of the European Society of Cardiology (ESC) [1].

Patients were excluded if they met any of the following criteria: severe renal impairment, a prior history of LAA thrombus, a history of cardioembolic stroke, known allergy to iodinated contrast media, presence of mechanical prosthetic valves, or an interval between CCT and TEE exceeding 24 h. Patients with different heart rhythms during CCT and TEE were excluded to standardize LAA hemodynamic conditions. Since LAA flow velocities and stasis patterns significantly differ between sinus rhythm and atrial fibrillation, maintaining a consistent rhythm across both imaging sessions are essential to ensure that diagnostic comparisons reflected modality performance rather than rhythm-induced changes in LAA blood flow. Patients with a history of stroke or TIA were included only if a cardioembolic etiology had been ruled out by a comprehensive neurological workup (e.g., carotid imaging and prior echocardiography). A history of confirmed cardioembolic stroke was maintained as a strict exclusion criterion to ensure the cohort focused on the primary detection of LAA thrombus prior to AF ablation. Additionally, patients with poor image quality on CCT that precluded definitive LAA assessment were excluded from the final analysis. From this initial pool, 102 patients were excluded: 24 due to severe renal impairment (eGFR < 30 mL/min/1.73 m²), 6 due to known contrast allergy, 24 because the interval between CCT and TEE exceeded the strict 24-hour limit, and 48 for other reasons including the presence of prosthetic cardiac valves, prior LAA closure devices (*n* = 28), or non-diagnostic CCT image quality due to severe motion artifacts (*n* = 20). This leaves a final study population of 705 patients.

Baseline demographics, clinical and imaging characteristics, and CHA_2_DS_2_-VASc score were obtained from patients’ files or electronic hospital records.

### Echocardiography

Left atrial size and LV ejection fraction were assessed by TTE prior to the TEE and CCT exams. TEE was performed using a GE Vivid E9 (GE Healthcare) ultrasound system equipped with 6VT-D probes. A complete 2D, colored, pulsed, and continuous-wave Doppler echocardiogram was performed according to EACVI recommendations [11]. The imaging was focused on the LA or LAA to attain optimal views and image quality. Three experienced cardiologists blinded to the CCT findings visually assessed the images for the presence of LAA thrombus. Thrombus in the LA or LAA was defined as a distinct intracavitary echo-dense mass that demonstrated acoustic characteristics distinct from the underlying endocardium. SEC (spontaneous echocardiographic contrast) was defined as slow swirling “smoke-like” echo densities and graded according to Fatkin et al. [12] Left atrial appendage velocity profiles were obtained by pulsed wave Doppler interrogation at the orifice of the appendage.

### CCT protocol

All patients underwent CCT using a dual-source multidetector CT scanner (Siemens Somatom Force, Siemens Healthineers, Germany) with retrospective ECG gating. Beta-adrenergic blocking agents were administered before the scan procedure if the heart rate was ≥ 80 bpm. An intravenous contrast agent (100 mL of iohexol, 300 mg iodine/ml) was injected using a power injector at a flow rate of 4 mL/s, followed by saline. Contrast-enhanced images were acquired during a single breath-hold using a bolus tracking technique with a threshold of 100 Hounsfield units in the ascending aorta. The scanning range was 1 cm below the carina to the heart base and diaphragm. The CT scan parameters were as follows: tube voltage of 100-120 kV, automated tube current modulation (CareDose 4D, Siemens Healthineers), detector collimation of 2 × 192 × 0.6 mm, gantry rotation time of 250 ms, a pitch of 0.2 and a matrix size of 512 × 512. Images were reconstructed with slice thickness of 0.6 mm and an increment of 0.4 mm.

The images were analyzed using dedicated software (CT Coronary, Syngo.via VB60A, Siemens Healthineers) by two experienced radiologists. The presence or absence of LAA thrombus, as well as the presence of any LAA types that could indicate a potential for thrombus formation, were evaluated. The LAA was classified according to Kimura et al. [13]. The LA was also evaluated for any other abnormal findings that may indicate a higher risk for thrombus formation, such as enlarged atrial dimensions or low contrast density within the left atrium.

The institutional protocol for patients undergoing pulmonary vein isolation (PVI) involves a standardized pre-procedural assessment. All patients underwent both CCT and TEE within a 24-hour window, regardless of the findings of either imaging modality. CCT was performed for the primary purpose of 3D anatomical mapping and pulmonary vein characterization, while TEE was performed as the mandatory gold standard for thrombus exclusion. This routine dual-imaging approach ensured that TEE was not selectively performed based on CCT results, thereby eliminating potential selection bias.

To ensure diagnostic consistency, a representative subgroup of 100 cases was independently reviewed by two blinded cardiologists. Inter-observer reliability for the detection of LAA thrombus was assessed using Cohen’s Kappa (kappa) statistics. The analysis showed excellent agreement for both TEE (kappa = 0.92) and CCT (kappa = 0.88).

### Statistical analysis

Statistical analyses were performed using IBM^®^ SPSS software version 28. Descriptive statistics were presented as frequency (percent), mean ± SD, or median (min-max). The χ^2^ and Exact tests were used to compare the proportions in different categorical groups. Continuous variables were investigated with visual and analytical methods to determine the normal distribution and analyzed with the Mann-Whitney U test or Student`s t-test where appropriate. The diagnostic predictive property of CCT in detecting LAA thrombus was analyzed by receiver operating characteristic (ROC) curve analysis. The area under the curve and the sensitivity and specificity of the test were calculated. An overall type-1 error level was used to infer statistical significance.

### Artificial intelligence

In this study, artificial intelligence was utilized exclusively to enhance the clarity and structure of English sentences, ensuring linguistic accuracy without influencing the scientific content or outcomes of the study.

## Results

### Baseline characteristics

Seven hundred five patients (50.6% male; mean age 57.9 ± 12.3 years) were included in the study, with a significant portion undergoing anticoagulation therapy 622 (88.2%). Most patients had paroxysmal AF (54.2%). Out of all patients, 128 (18.2%) had a CHA₂DS₂-VASc score of 0, 207 (29,4%) had a CHA₂DS₂-VASc score of 1, and 370 (47,6%) had a CHA₂DS₂-VASc score ≥ 2. Other baseline characteristics of the entire study population are presented in Table 1.

**Table 1:** Clinical characteristics of patient population(*n* = 705)

Age, years	57.8 ± 12.3
Male sex	357 (50.6%)
OAC	622 (88.2%)
Paroxysmal AF	377 (54.2%)
Hypertension	364 (51.6%)
Coronary artery disease	288 (40.9%)
LV dysfunction/CHF	173 (24.5%)
Diabetes mellitus	140 (19.9%)
Peripheral artery disease	40 (5.7%)
Prior stroke/TIA	32 (4,5%)
LA size, mm	40 ± 5.7
LV ejection fraction %	60 (19–72)
CHA_2_DS_2_-VASc score	2 (0–7)
CHA_2_DS_2_-VASc score 0	128 (18.2%)
CHA_2_DS_2_-VASc score 1	207 (29.4%)
CHA_2_DS_2_-VASc score 2	145 (20.6%)
CHA_2_DS_2_-VASc score 3	137 (19.4%)
CHA_2_DS_2_-VASc score ≥ 4	88 (12.5%)

CHA2DS2-VASc — congestive heart failure, hypertension, age ≥75, diabetes, stroke, vascular disease, age 65 — 74, sex category

*AF* atrial fibrillation, *CHF* congestive heart failure, *LA* left atrial, *LV* left ventricular, *OAC* oral anticoagulant, *TIA* transitory ischemic attack

#### TEE and CCT

**Fig. 1:**
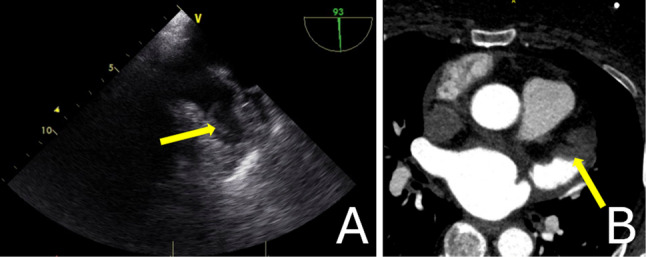
TEE and cardiac CT images from a 62-year-old woman with AF and a LAA thrombus. **A** TEE demonstrated a thrombus (arrows) with SEC. **B** CT demonstrated an oval-shaped filling defect in the LAA (arrows) suggestive of thrombus

All 705 patients in the study underwent both CCT and TEE with intervals of less than 1 day between the two procedures. In all cases, image quality was technically adequate for clinical assessment. Out of 705 patients, 1.8% (13 patients) had LA/LAA thrombus detected by TEE, while 7% (49 patients) had SEC without thrombus Table 2. Among the 13 patients identified with LAA thrombus, all were receiving oral anticoagulation (OAC) therapy at the time of the diagnosis. The distribution of OAC regimens was as follows: 3 patients were on Warfarin (all within the target INR range of 2.0–3.0), 4 patients were on Apixaban (three on 5 mg bid, one on 2.5 mg bid), 3 patients were on Rivaroxaban (20 mg od), and 3 patients were on Edoxaban (60 mg od). In all 13 cases where LAA thrombus was confirmed by TEE, the scheduled pulmonary vein isolation procedure was cancelled. Patient adherence to oral anticoagulation (OAC) was thoroughly reassessed; three patients identified as non-adherent were strictly counseled on medication compliance, and follow-up TEE was scheduled 6 weeks later. Notably, in patients who were found to have persistent LAA thrombus despite optimized and adherent OAC therapy, percutaneous LAA closure was performed as an alternative strategy [14]. Among the 49 patients with SEC, 1.7% (12 patients) had mild, 3.3% (23 patients) had moderate, and 2% (14 patients) had severe SEC. The diagnostic performance of CCT and TEE showed significant systematic discordance according to the McNemar test (*p* < 0.001). While CCT excluded thrombus in 583 of 584 cases confirmed negative by TEE (high NPV), it yielded 109 false-positive results. A detailed analysis of diagnostic discordance revealed that the presence of SEC on TEE was a major predictor of false-positive CCT results. Out of 49 patients with SEC, 33 (67.3%) had corresponding filling defects on CCT (*p* < 0.001; Phi coefficient = 0.245). In contrast, a filling defect was observed in the LAA in 121 out of 705 cardiac CT scans (17.2%), suggesting the possibility of a thrombus that could not be ruled out Table 2. Figure 1. gives an example of an LAA thrombus, visualized by both TEE and CCT.

**Table 2:** Comparison of CCT and TEE imaging results for detection of left atrial appendage thrombus and sec in a population of 705 patients

TEE thrombus	13 (1.8%)
SEC in LAA total	49 (7%)
Grade-1	12 (1.7%)
Grade-2	23 (3.3%)
Grade-3	14 (2%)
CCT filling defect	121 (17.2%)

*CCT* cardiac computed tomography, *TEE* transesophageal echocardiography, *SEC* spontaneous echo contrast

**Fig. 2:**
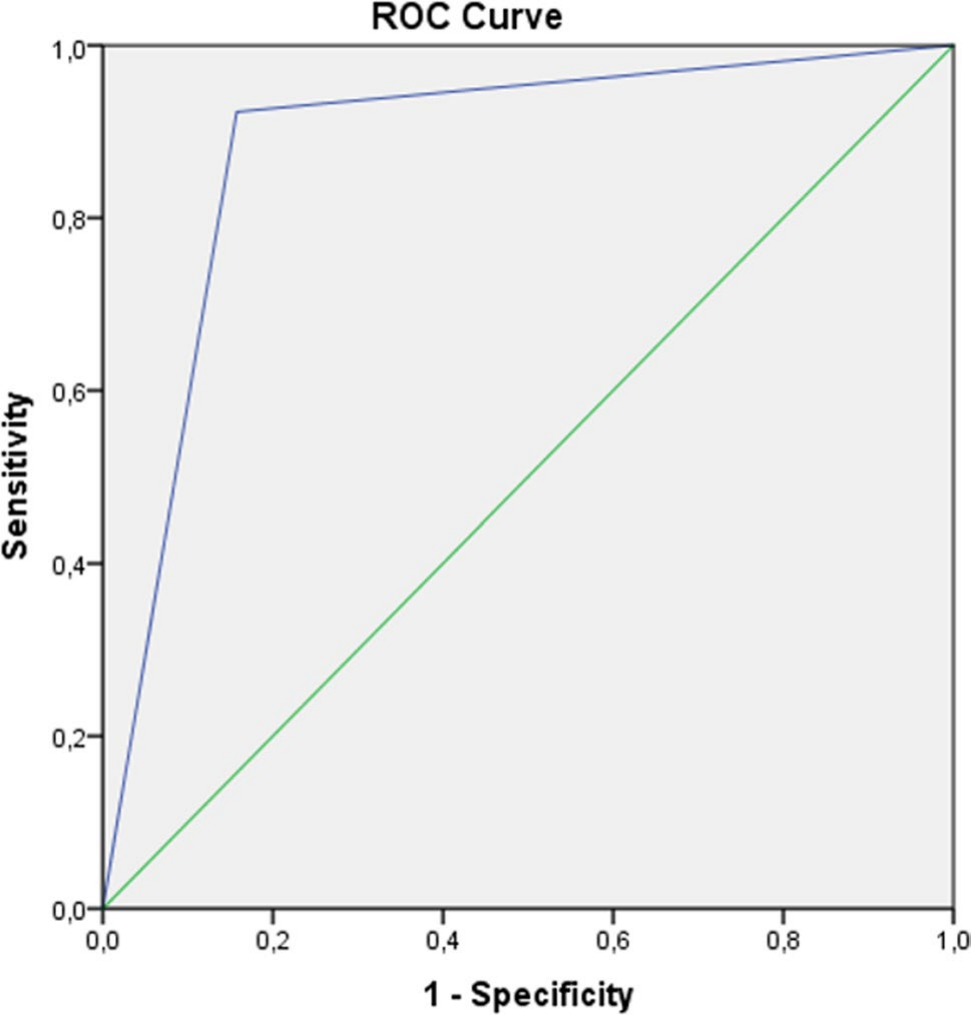
Receiver operating characteristics curve the diagnostic predictive property of CCT in detecting LAA thrombus. Sensitivity = 92.3%, specificity = 84.2%, area under the receiver operating characteristic curve 0.883 (95% CI: 0.797–0.969; *p* = 0,001)

Using TEE as the reference standard, the overall sensitivity, specificity, PPV, and NPV of CCT for thrombi detection in the LAA were 92.3%, 84.2%, 9.9% and 99.8%, respectively. Table 3; Fig. 2 .

**Table 3:** Diagnostic accuracy of CCT compared to TEE for detection of LA/LAA thrombus

Sensitivity	92.3%
Specificity	84.2%
NPV	99.8%
PPV	9.9%

*CCT* cardiac computed tomography, *TEE* transesophageal echocardiography, *LAA* left atrial appendage

### Factors associated with LA/LAA thrombus

**Fig. 3:**
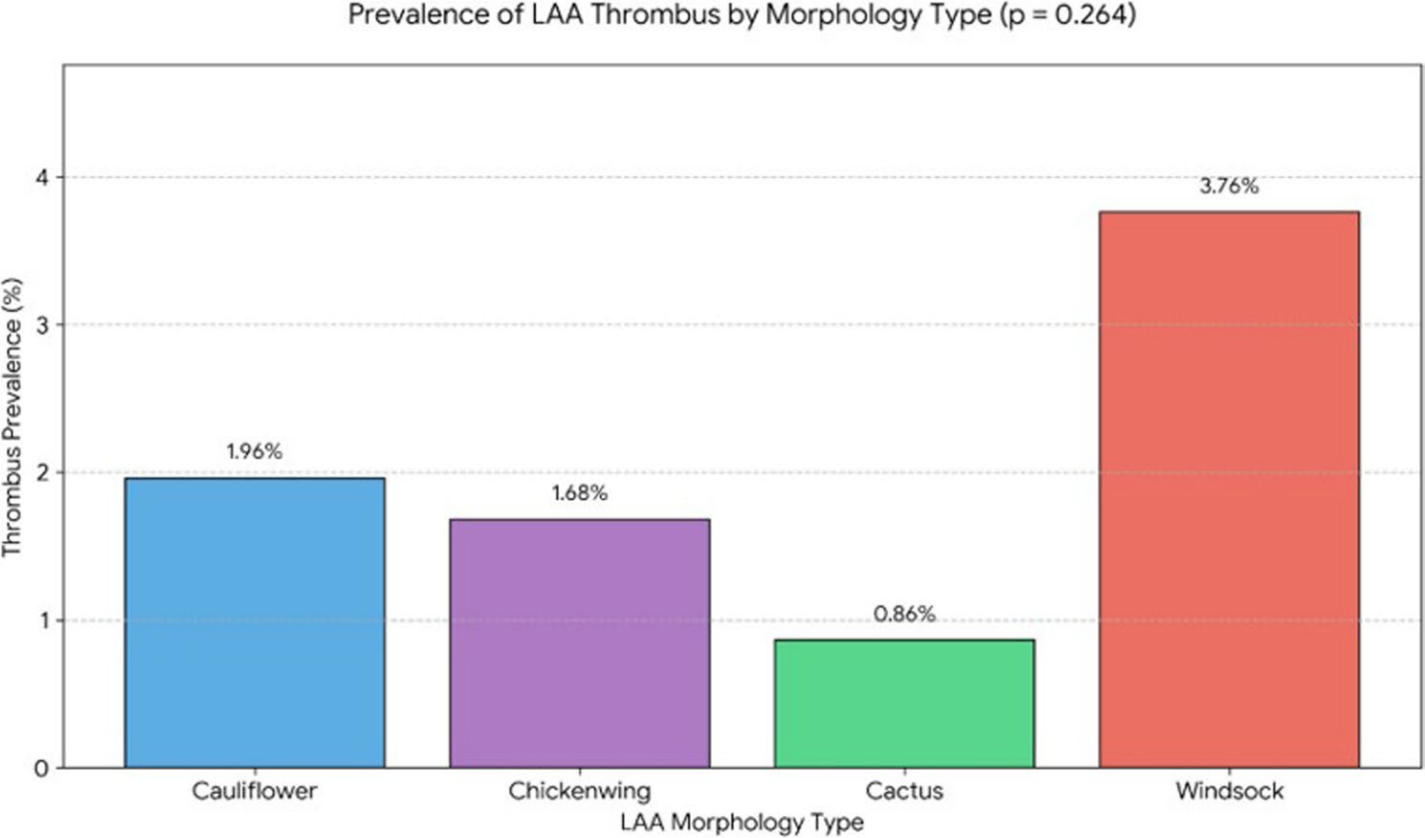
The distribution of LAA types detected by CCT according to the presence of thrombus in the left atrial appendage on TEE

Regarding LAA anatomy, although the Windsock morphology was numerically more frequent in patients with thrombus, this relationship did not reach statistical significance Table 4; Fig. 3.

**Table 4:** Factors associated with LAA thrombus

Parameters, mean ± Sd	LAA thrombus		*p* value (95% CI)
	Presence of LAA thrombus	No LAA-thrombus	
	*n*=13	*n*=692	
LAA types *n* (%)			0.264
cauliflower	2 (15.4 %)	100 (14.5%)	
chicken wing	4 (30.8 %)	234 (33.8 %)	
cactus	2 (15.4 %)	230 (33.2%)	
windsock	5 (38.5 %)	128 (18.5 %)	
LAA emptying velocity, m/sec	0.21 ±0.1	0.56 ± 0.2	0.018 (-0.64, -0.05)
LA size, mm	43.5 ±6.3	40.1 ±5.7	0.03 (0.33, 6.63)
CHA_2_DS_2_-VASc score	3 (0-6)	2 (0-7)	0.117

CHA2DS2-VASc — congestive heart failure, hypertension, age ≥75, diabetes, stroke, vascular disease, age 65 — 74, sex category

*LA* left atrial, *LAA* left atrial appendage, (95% *CI* confidence interval for mean/median difference between groups.)

Furthermore, specific parameters such as LAA emptying velocity and LA size were assessed. The mean LAA emptying velocity was 0.21 ± 0.1 m/sec in patients with LAA thrombus, which was significantly lower than the velocity in patients without LAA thrombus (0.56 ± 0.2 m/sec; *p* = 0.018). Additionally, patients with LAA thrombus exhibited a larger LA size (43.5 ± 6.3 mm) compared to those without LAA thrombus (40.1 ± 5.7 mm; *p* = 0.03). The comparison of CHA₂DS₂-VASc scores between patients with LAA thrombus (median: 3; range: 0–6) and those without LAA thrombus (median: 2; range: 0–7) did not yield statistical significance (*p* = 0.117).

Multivariable logistic regression analysis was performed to identify independent predictors of LAA thrombus, incorporating LAA flow velocity and LA diameter into the model. The model demonstrated an excellent fit (Hosmer-Lemeshow test, *p* = 0.99) and revealed that LAA peak flow velocity was the only significant variable associated of thrombus presence (B: -9.335, *p* = 0.021; 95% CI for Exp(B): 0.000–0.252).

## Discussion

The main finding of our trial is that CCT has a high sensitivity (92.3%) and negative predictive value (99.8%) for detecting LAA thrombus, indicating that CCT is a reliable tool for ruling out the presence of thrombus in the LAA. However, the specificity (84.2%) and positive predictive value (9.9%) were relatively low, suggesting that CCT may not be as reliable in detecting the presence of LAA thrombus.

The diagnostic results of CCT for predicting LAA thrombus before CA for AF were previously evaluated. Our findings are consistent with previous studies that have reported higher sensitivity but lower specificity for CCT compared to TEE in detecting LAA thrombus [8, 9, 15, 16]. Kim YY et al. [8] used 16, 40, and 64-slice multidetector computed tomography (MDCT) in their trial, while Tang RB et al. [9] and Martinez MW et al. [14] used 64-slice MDCT. The lower specificity of CCT in their trial can be attributed mainly to sliced-related image quality differences.

The diagnostic performance of CCT in our study showed a very low PPV (9.9%), which is primarily attributable to the single-phase acquisition protocol. In the absence of a delayed phase, contrast opacification within the LAA may be incomplete due to slow flow or stasis—a phenomenon often seen in patients with AF—leading to false-positive ‘filling defects.’ Romero et al. [17] demonstrated that delayed imaging (at 60 s) significantly improves the PPV of CCT by allowing contrast to eventually fill the LAA in cases of stasis, thereby differentiating it from true thrombus. Similarly, Korsholm et al. [18] reported that a dual-phase CCT protocol could potentially match the diagnostic accuracy of TEE. In our cohort, we deliberately avoided a delayed phase to minimize radiation exposure, adhering to the ALARA principle, especially since TEE was routinely performed for all patients. However, we acknowledge that this represents a trade-off between diagnostic specificity and radiation safety. Future clinical workflows might benefit from AI-integrated systems that can analyze Hounsfield Unit (HU) gradients or flow dynamics to predict thrombus without the need for additional radiation, as suggested by recent perspectives on the role of AI in cardiology [19]. Specifically, AI algorithms excel in pattern recognition and can detect subtle abnormalities in cardiac imaging that might escape human observation.

The clinical significance of SEC as a confounder for CCT accuracy is clearly demonstrated in our results. Specifically, 33 out of 49 patients (67.3%) with SEC on TEE showed corresponding filling defects on CCT, all of which were confirmed to be false positives. This confirms that high-grade stasis (sludge) significantly alters LAA opacification during the single arterial phase, mimicking true thrombus. The strong correlation we observed between SEC and CT filling defects adds to the growing body of evidence that these imaging modalities, while utilizing different physical principles, are detecting complementary aspects of the same thrombogenic process. This finding has important implications for the development of integrated imaging algorithms for LAA thrombus risk assessment. Limitations of this analysis include the retrospective design and the use of single-phase CT acquisition. The latter limitation is particularly relevant, as delayed-phase imaging might have reduced the number of false-positive filling defects, potentially revealing an even stronger correlation between true blood stasis (SEC) and persistent CT abnormalities [20]. Future prospective studies utilizing dual-phase CT protocols should examine whether the combination of SEC on TEE and persistent filling defects on delayed CT identifies a particularly high-risk phenotype for thromboembolic events.

As our study confirms, TEE remains the gold standard for identifying LAA thrombus [5, 21]. TEE has the advantage of high temporal and spatial resolution and the lack of radiation and contrast exposure. However, TEE presents several drawbacks, including its high cost, invasiveness, reliance on operator skill, susceptibility to interobserver variability, and the potential for causing discomfort and severe complications such as esophageal injury, perforation, or aspiration [22]. The clinical integration of CCT as a primary screening tool for LAA thrombus involves a strategic trade-off. From a cost-efficiency standpoint, the low PPV (9.9%) of single-phase CCT means that nearly one-fifth of our cohort required a follow-up TEE, potentially increasing total diagnostic costs and time. However, this must be balanced against the benefit for the remaining 82.8% of patients, in whom CCT confidently excluded thrombus, thereby sparing them from an invasive TEE procedure. In centers where CCT is routinely performed for pulmonary vein mapping, utilizing it as a robust exclusion filter may be more patient-centric, although TEE remains a more direct and efficient strategy in resource-limited settings or where procedural speed is prioritized. Furthermore, clinical decision-making must also account for the inherent risks of CCT, including radiation exposure and the potential for contrast-induced nephropathy. While TEE avoids these issues, it carries its own procedural risks as an invasive modality. A balanced clinical strategy should therefore weigh these technology-specific complications against the high negative predictive value and patient comfort provided by a non-invasive CCT screening approach.

The low prevalence of LAA thrombus observed in our study (1.8%) is consistent with recent reports in the era of non-vitamin K antagonist oral anticoagulants (NOACs) [23]. While this low prevalence inherently leads to a lower PPV, it highlights the exceptional clinical utility of CCT’s Negative Predictive Value (NPV). In clinical practice, the primary goal of pre-ablation imaging is to identify patients in whom the procedure can safely proceed. Our findings confirm that a negative CCT provides near-certainty regarding the absence of thrombus, supporting its role as a primary screening tool. This high NPV, validated in a large-scale cohort with a strict 24-hour imaging interval, offers practical reassurance to clinicians that TEE can be reserved for patients with ambiguous CCT findings, thereby optimizing procedural workflows and patient comfort.

CCT is a valuable imaging modality for assessing the LAA in patients with AF. It is non-invasive, well-tolerated, and provides detailed information about LAA anatomy and function. In addition, it can be used as an alternative to TEE in patients who are unable to tolerate TEE or have contraindications to the procedure. Compared to TEE, CCT has several advantages, including its noninvasive nature, shorter examination time, and lower risk of complications.

The mean LAA emptying velocity was significantly lower in patients with LAA thrombus (0.21 ± 0.1 m/sec) compared to those without LAA thrombus (0.56 ± 0.2 m/sec) (*p* = 0.018). This finding is consistent with previous studies that have shown a lower LAA emptying velocity in patients with LAA thrombus [24]. A reduced LAA emptying velocity can lead to blood stasis in the LAA, which in turn can promote thrombus formation.

Similarly, patients with LAA thrombus had a larger LA size (43.5 ± 6.3 mm) compared to patients without LAA thrombus (40.1 ± 5.7 mm) (*p* = 0.03). The LA size is known to be an important predictor of LAA thrombus, as an enlarged LA can lead to blood stasis and promote thrombus formation [24].

Even though the CHA₂DS₂-VASc score is the most used and suggested tool for predicting stroke risk in AF patients, did not show a significant difference between patients with and without LAA thrombus (*p* = 0.117). This result is somewhat unexpected, given the established association between higher CHA₂DS₂-VASc scores and increased stroke risk in AF, frequently linked to LAA thrombus. Nevertheless, the study’s small sample size of patients with LAA thrombus may have influenced the lack of statistical significance in the CHA₂DS₂-VASc scores. It is important to note that the CHA₂DS₂-VASc score is primarily validated for predicting long-term clinical thromboembolic events rather than the acute presence of LAA thrombus on imaging. This conceptual distinction may explain the lack of a significant correlation in our study. While the score remains the gold standard for long-term anticoagulation decisions, our findings suggest that acute thrombogenesis may be more closely linked to local hemodynamic factors—such as low LAA flow velocity—than to the cumulative clinical risk factors represented by the CHA₂DS₂-VASc score.

Our study has several limitations. Firstly, the sample size of patients with LAA thrombus identified in TEE is relatively small, necessitating larger-scale trials for more definitive conclusions. Secondly, the study’s retrospective design limits its ability to accurately reflect the clinical significance of SEC and the incidence of false positive thrombus formations in CCT. Thirdly, a cost-effectiveness analysis was not conducted in our trial. Lastly, the relatively small number of confirmed LAA thrombus cases (*n* = 13) is a notable limitation. The low event rate affects the precision of all diagnostic estimates, as reflected by the wide AUC confidence interval (0.797–0.969), and renders the multivariable logistic regression model exploratory rather than confirmatory. Consequently, the identified associated variables should not be regarded as definitive independent predictors, and larger prospective studies are warranted to validate these findings.

## Conclusion

Our study provides compelling evidence that CCT is a reliable imaging technique for excluding the existence of thrombus in the LAA among patients with symptomatic paroxysmal or persistent AF who are scheduled for PVI. Our results suggest that for AF patients with adequate anticoagulation, if CCT demonstrates no thrombus, then catheter ablation and DCCV procedures can be safely performed.

## Supplementary Information

Supplementary Material 1.

## Data Availability

The datasets used and/or analyzed during the current study are available from the corresponding author upon reasonable request.
