# Supplementary material for: Comparison of cardiac computed tomography and transesophageal echocardiography for left atrial appendage thrombus detection
Source: BMC Cardiovasc Disord. 2026 May 29;26:649. doi: 10.1186/s12872-026-06012-3 (PMC13421734; doi:10.1186/s12872-026-06012-3)
Supplement: Supplementary file 1 — Supplementary Material 1. [file 12872_2026_6012_MOESM1_ESM.docx]

Cardiac computed tomography demonstrates high sensitivity (92.3%) and negative predictive value (99.8%) for ruling out left atrial appendage thrombus in patients with atrial fibrillation. With adequate anticoagulation, it provides a safe non-invasive screening alternative to transesophageal echocardiography prior to pulmonary vein isolation procedures.
